# Comparison of ocular biometry and refractive outcomes using two swept-source optical coherence tomography-based biometers

**DOI:** 10.1371/journal.pone.0316439

**Published:** 2024-12-31

**Authors:** Hansol Park, Young-Sik Yoo, Eunhae Shin, Won Seok Song, Yeokyoung Won, Tae-Young Chung, Dong Hui Lim

**Affiliations:** 1 Department of Ophthalmology, Samsung Medical Center School of Medicine, Sungkyunkwan University, Seoul, Republic of Korea; 2 Department of Ophthalmology, College of Medicine, The Catholic University of Korea, Seoul, Republic of Korea; 3 Renew Seoul Eye Center, Seoul, Republic of Korea; 4 Samsung Advanced Institute for Health Sciences & Technology, Sungkyunkwan University, Seoul, Republic of Korea; Saarland University, GERMANY

## Abstract

**Background:**

To evaluate the ocular biometry agreement and prediction of postoperative refractive outcomes obtained using two swept-source optical coherence tomography (SS-OCT) biometers: Anterion (Heidelberg Engineering, Heidelberg, Germany) and Argos (Alcon, Fort Worth, TX, USA).

**Methods:**

Ambispective analysis was conducted on 105 eyes at the Samsung Medical Center, Seoul, Republic of Korea, between June 2021 and March 2022. Biometric values were assessed using both devices before cataract surgery. Intraocular lens (IOL) power, mean arithmetic error (ME), and mean absolute error (MAE) were calculated using the Barrett Universal II, Haigis, and Hoffer Q formulas.

**Results:**

Anterion showed statistically significantly greater axial length (AL), central corneal thickness (CCT), and lens thickness (LT) than Argos (p = 0.03, p < 0.001, and p = 0.032, respectively). There were no significant differences in measuring anterior chamber depth (ACD) (p > 0.05). Anterion showed flatter corneal curvature measurements than Argos (p < 0.001). The postoperative prediction errors differed for all three formulas (p < 0.001). Anterion results leaned towards a slightly myopic outcome due to hyperopic target refraction. In all three formulas, the MAE and percentage of eyes with a prediction error ≤ ± 0.5 D were not significantly different between the two devices.

**Conclusion:**

Although the differences are not clinically significant, the measurements of AL, CCT, and LT obtained with Anterion were greater compared to those measured with Argos, while the keratometry (K) and corneal diameter (CD) values were smaller. Consequently, this resulted in a minor difference in refractive predictability, with Anterion showing a slight tendency toward more myopic refractive errors. However, there were no significant differences in MAE or the percentage of eyes within ± 0.5D.

## Introduction

With advances in cataract surgery techniques, the emphasis has transcended from mere opacification removal to precisely correct refractive errors. Consequently, the accuracy of preoperative ocular measurements is critical for successful cataract intervention. The corneal topography, axial length (AL), and anterior chamber depth (ACD) are important parameters in intraocular lens (IOL) calculation. Earlier studies indicated that a 1.0-mm discrepancy in AL and ACD measurements can lead to refractive errors of 2.7 and 1.5 diopters (D), respectively [1]. Technologies for precise ocular measurements are constantly evolving; instruments incorporating these advancements are continuously being introduced. The Orbscan and Scheimflug camera-based Pentacam were primarily used to obtain corneal information. Trends in ocular measurements, notably AL, have transitioned from ultrasound-based to predominantly partial coherence interferometry (PCI)-based methods. However, recent reports suggested that computerized corneal topography and ocular measurements using Optical Coherence Tomography (OCT) have improved the accuracy of preoperative ocular measurements [2,3].

The Anterion (Heidelberg Engineering Inc., Heidelberg, Germany), a novel swept source OCT (SS-OCT) device, can capture a broader scan depth (14.5 mm) and width (16.5 mm). The device has improved the image quality of anterior segment tomography by reducing scattering, which has been identified as a drawback in conventional OCT, while simultaneously increasing sensitivity. It employs a light source with a wavelength of 1300 nm and boasts a high axial resolution < 10 um [4]. However, there are limited reports on the utility of anions in calculating the power of IOL.

Argos (Alcon, Fort Worth, TX, USA) is another SS-OCT-based device that utilizes a 1060-nm wavelength to record two-dimensional OCT images from the corneal apex to the macula. AXL, ACD, lens thickness (LT), and central corneal thickness (CCT) were calculated using the OCT images. This study aimed to compare the biometric measurements and refractive outcomes of two SS-OCT-based devices (Anterion and Argos), which can simultaneously perform computerized corneal topography and ocular measurements.

## Methods

### Data source and study setting

This ambispective study included patients who underwent phacoemulsification and IOL insertion in the Anterior Segment Unit of Samsung Medical Center, Seoul, Republic of Korea between June 2021 and March 2022. First, pre- and postoperative data were prospectively collected from 25 patients who provided informed written consent. Due to recruitment challenges during the COVID-19 pandemic, data collection and analysis from the remaining 80 patients were conducted retrospectively, thus obviating the need for patient consent. Patients with preexisting conditions such as corneal or retinal diseases and glaucoma, which can make accurate eye measurements difficult, were excluded. Additionally, patients who experienced surgical complications, such as zonulysis or posterior capsule rupture, which could potentially affect the accuracy of refractive error assessments, were also excluded. We further narrowed the scope to patients who received the Acrysof SN60WF IOL (Alcon Laboratories, Inc., Fort Worth, TX, USA).

A total of 105 eyes were enrolled; of these, 57 were from single-eye patients and 24 were from those in whom both eyes were treated. The mean patient age was 71.43±10.82 years; 58 (55.2%) patients were female. All patients underwent ocular biometric measurements with Argos and Anterion before cataract surgery. Each device had three examiners who performed the measurements in a randomized sequence. These measurements were performed prior to pupil dilation, following the manufacturer’s guidelines. All surgical procedures were performed by a single surgeon (TY Chung) using a temporal near-clear incision 2.75 mm in size. Comprehensive ophthalmological examinations, including visual examination, were also performed on all enrolled patients. An acuity test, tonometry, and fundus examination were performed before and after surgery. One month postoperatively, the best-corrected visual acuity (BCVA, expressed as a decimal value) and manifest refraction (MR) were evaluated.

This study was approved by the Institutional Review Board (IRB number: 2020-04-052) of the Samsung Medical Center, Seoul, Korea. All study methods adhered to the tenets of the Declaration of Helsinki.

### Instruments

Argos, an SS-OCT-based biometer with 1060-nm laser infrared light, utilizes reflection keratometry and derives measurements from the reflections of 16 points located on a concentric circle with a diameter of 2.2 mm. The instrument captures three OCT images for each acquisition, thereby allowing the measurement of various parameters, including AL, ACD, central corneal thickness (CCT), aqueous depth, lens thickness (LT), pupil size, and corneal diameter (CD). AL was calculated based on standard refractive indices: 1.376 for the cornea, 1.336 for the aqueous and vitreous humors, and 1.410 for the crystalline lens. The final biometric values were calculated as the average of five individual measurements per each segment [5].

The Anterion uses a 1300-nm wavelength light source and measures keratometry values with a total of 65 radial B-scan images in the 3-mm zone of the central cornea. AL is determined by using a single refractive index that is applicable to the entire eye. The device also offers comprehensive tomographic assessments of the cornea, capturing both posterior and total corneal powers, which can be integrated into certain IOL formulas. Biometric values were derived from a single measurement [6].

Measurements of AL and simulated keratometry values (K1 for the flattest axis and K2 for the steepest axis, separated by 90°) of the anterior corneal surface, ACD, LT, and CD were also documented.

### IOL power calculation

For each device, the IOL power was determined using three formulas: Barrett Universal II, Haigis, and Hoffer Q. To determine the refractive prediction error (PE), the predicted spherical refraction based on the IOL was subtracted from the spherical equivalent (SE) of subjective refraction. Metrics to evaluate the precision of the IOL power estimation included the standard deviation (SD) of the PE, the mean arithmetic PE (ME), the mean absolute PE (MAE), and the proportion of eyes with a PE within ± 0.5 D.

### Statistical analysis

Data were analyzed using SAS version 9.4 (SAS Institute Inc., Cary, NC, USA). For pre- and postoperative statistical comparisons, a paired t-test was used for variables that conformed to a normal distribution. The non-parametric signed-rank test was used for variables that deviated from a normal distribution. To account for the potential correlation between measurements from both eyes of the same patient, a generalized estimating equations (GEE) approach was additionally employed. Furthermore, McNemar’s test was conducted to evaluate the attainment of postoperative refractive outcomes within a ± 0.5-D range of the target. The degree of inter-device agreement was evaluated using Bland–Altman plots [7]. The 95% limit of agreement (LoA) was defined as the mean ± 1.96 SD of the difference between paired devices. A *p* value < 0.05 was considered statistically significant.

## Results

### Biometry parameter agreement

**Fig 1:**
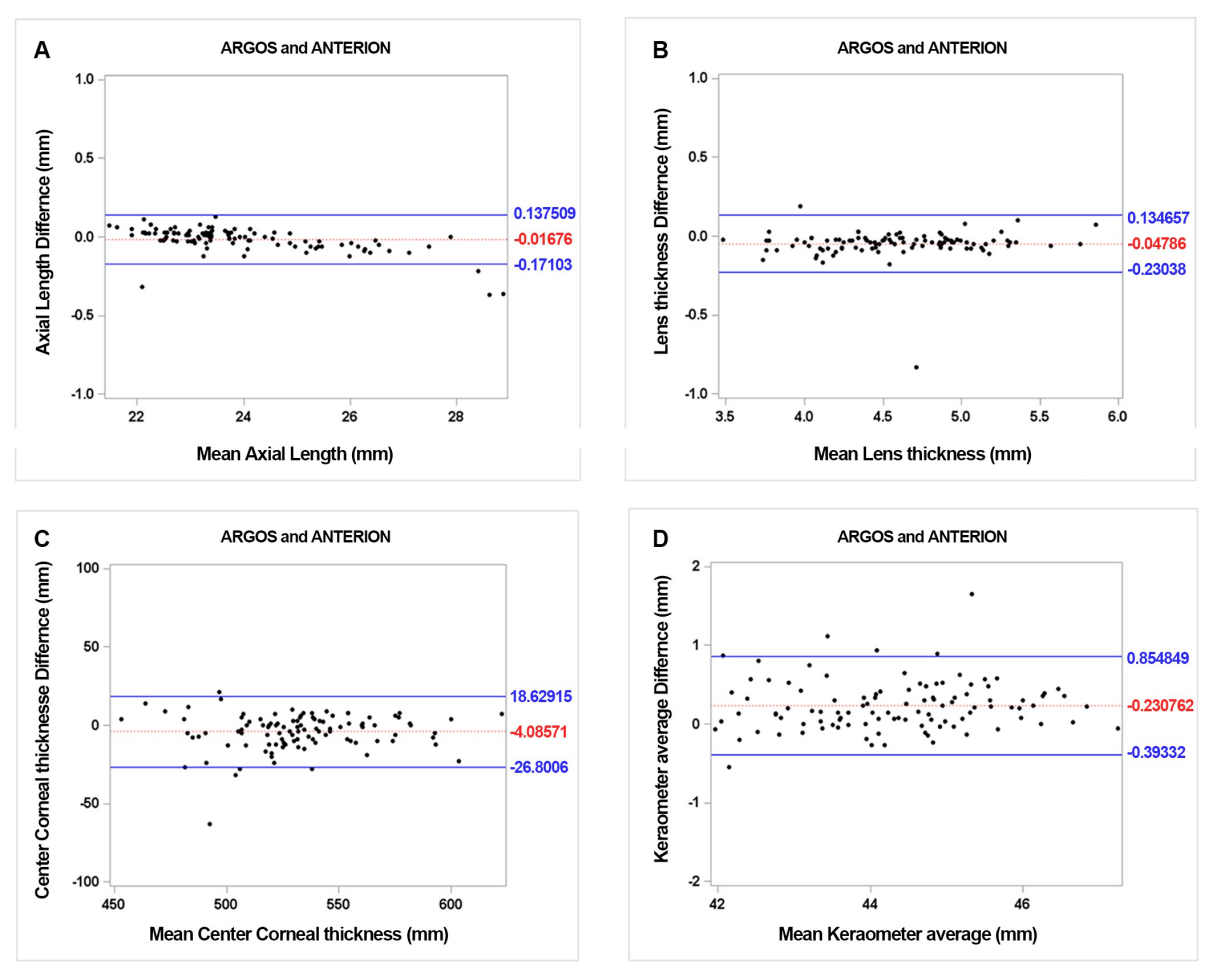
Bland-Altman plot of biometric values for two SS-OCT devices. The mean difference is indicated by the dotted lines; 95% LoA is indicated by the solid line. AL (A); LT (B); CCT (C); and Kav (D).

The mean preoperative AL was 23.89 ± 1.58 mm for Argos and 23.91 ± 1.63 mm for Anterion, which demonstrated statistically significant difference (Table 1, p = 0.03). The 95% LoA for AL ranged from -0.17 to 0.14 (p = 0.16). All values except those from four eyes were within this range. (Fig 1). The ACD measurements of the two devices were not statistically different. (p = 0.11).

**Table 1:** Biometry values for Argos and Anterion.

	Argos		Anterion		Mean difference	P value	P value^†^ by GEE
	Mean ± SD	Range	Mean ± SD	Range			
AL (mm)	23.89 ± 1.58	21.51 ∼ 28.69	23.91 ± 1.63	21.44 ∼ 29.05	0.02	0.1611^b^	0.028
CCT (um)	530.33 ± 32.13	455 ∼ 626	534.42 ± 31.31	451 ∼ 619	4.09	<0.0001^a^	<0.001
ACD (mm)	3.17 ± 0.45	2.2 ∼ 4.25	3.14 ± 0.48	2.15 ∼ 4.89	-0.03	0.1123^a^	0.108
LT (mm)	4.58 ± 0.49	3.47 ∼ 5.89	4.61 ± 0.47	3.49 ∼ 5.82	0.03	<0.0001^a^	0.032
CD (mm)	11.98 ± 0.53	10.1 ∼ 13.38	11.48 ± 0.41	10.53 ∼ 12.92	-0.5	<0.0001^a^	<0.001
Kav (D)	44.43 ± 1.26	41.87 ∼ 47.22	44.2 ± 1.23	41.63 ∼ 47.27	-0.23	<0.0001^a^	<0.001
K1 (D)	43.88 ± 1.27	41.0 ∼ 46.78	43.74 ± 1.24	40.89 ∼ 46.48	-0.14	<0.0001^a^	<0.001
K2 (D)	45.0 ± 1.32	42.32 ∼ 47.67	44.67 ± 1.29	42.03 ∼ 48.09	-0.33	<0.0001^a^	<0.001

SD, standard deviation; GEE, generalized estimating equations; AL, axial length; CCT, central corneal thickness; ACD, anterior chamber depth; LT, lens thickness; CD, corneal diameter; Kav, average keratometric value; K1, flat corneal curvature; K2, steep corneal curvature.

^a^Paired t-test

^b^Signed rank test

†P value generated from generalized estimating equations.

The average CCT, when measured with Anterion, was notably thicker at 534.42 ± 31.31 um, in contrast to 530 ± 32.13 um as determined by Argos, with a statistically significant difference (p < 0.001). Regarding the mean LT, Anterion also showed significantly thicker value at 4.61 ± 0.47 mm, than did the Argos at 4.58 ± 0.49 mm (p = 0.032). This difference was demonstrated using the Bland-Altman plot (Fig 1).

For comparison of CD, the values were 11.98 ± 0.53 mm and 11.48 ± 0.41 mm with Argos and Anterion, respectively. Argos showed a significantly longer CD than the anion (p < 0.001). In case of corneal curvature, Anterion showed generally flatter measurements as follows: Anterion, K1:43.74 ± 1.24 D, K2:44.67 ± 1.29 D, Kav: 44.2 ± 1.23 D; Argos, K1:43.88 ± 1.27 D, K2:45.0 ± 1.32 D, Kav: 44.43 ± 1.26 D. All values demonstrated statistically significant differences (p < 0.001). The Bland-Altman plot of Kav is shown in Fig 1.

### Postoperative refractive outcome

Among 105 eyes, postoperative prediction errors were measured in 100 eyes. Table 2 illustrates a comparison of the target diopters with the corresponding IOL diopters calculated according to the following formulas: Barrett Universal II, Hoffer Q, and Haigis. The differences in the target diopters calculated with all three formulas using Argos and Anterion were statistically significant (p < 0.001). The target diopters of the Anterion tended to be relatively hyperopic as compared to those of the Argos.

**Table 2:** Mean arithmetic error and mean absolute error of IOL power calculation using data from Argos and Anterion.

		Argos		Anterion		P value	P value† by GEE
		Mean ± SD	Range	Mean ± SD	Range		
**Barrett**	Target diopters	-0.92 ± 1.19	-4.93 ∼ 0.04	-0.79 ± 1.31	-4.59 ∼ 1.17	<0.0001^b^	<0.001
	ME (D)	0.13 ± 0.43	-0.97 ∼ 1.63	-0.09 ± 0.49	-1.17 ∼ 1.62	<0.0001^a^	<0.001
	MAE (D)	0.35 ± 0.29	0.01 ∼ 1.63	0.38 ± 0.32	0 ∼ 1.62	0.675^b^	0.352
	Eyes within ± 0.5D (%)	77.0		68.0		1.939^c^	
**Hoffer Q**	Target diopters	-0.88 ± 1.24	-5.06 ∼ 0.27	-0.73 ± 1.42	-4.98 ∼ 1.27	<0.0001^b^	<0.001
	ME (D)	0.08 ± 0.49	-2 ∼ 1.22	-0.04 ± 0.55	-1.22∼1.62	0.003^a^	0.002
	MAE (D)	0.37 ± 0.32	0 ∼ 2	0.42 ± 0.36	0∼1.62	0.220^b^	0.245
	Eyes within ± 0.5D (%)	70.0		72.0		0.033^c^	
**Haigis**	Target diopters	-0.92 ± 1.26	-5.21 ∼ 0.33	-0.73 ± 1.36	-4.66 ∼ 1.16	<0.0001^b^	<0.001
	ME (D)	0.13 ± 0.45	-0.95 ∼ 1.38	-0.04 ± 0.54	-1.12 ∼ 1.4	<0.0001^a^	<0.001
	MAE (D)	0.37 ± 0.28	0 ∼ 1.38	0.42 ± 0.33	0 ∼ 1.4	0.326^b^	0.130
	Eyes within ± 0.5D (%)	75.0		68.0		1.333^c^	

SD, standard deviation; ME, mean arithmetic prediction error; MAE, mean absolute prediction error; D, diopters.

^a^Paired t-test

^b^Signed rank test

^c^McNemar’s test

†P value generated from generalized estimating equations.

ME, MAE, and percentage of eyes with a prediction error within ± 0.5 D are also demonstrated in Table 2. The ME of Argos and Anterion were 0.13 ± 0.43 D and -0.09 ± 0.49 D, respectively, according to Barrett Universal II formula (p < 0.001). When it comes to the Hoffer Q and Haigis formula, the values were 0.08 ± 0.49 D vs. -0.04 ± 0.55 D (p = 0.002) and 0.13 ± 0.45 D vs. -0.04 ± 0.54 D (p < 0.001), respectively (Argos vs. Anterion). The refractive outcome of the Anterion was slightly myopic as compared to that of the Argos.

In all three formulas, the MAE and the percentage of eyes with a prediction error ≤ ± 0.5 D of Argos and Anterion were not statistically significantly different between the two devices (all p > 0.05).

**Fig 2:**
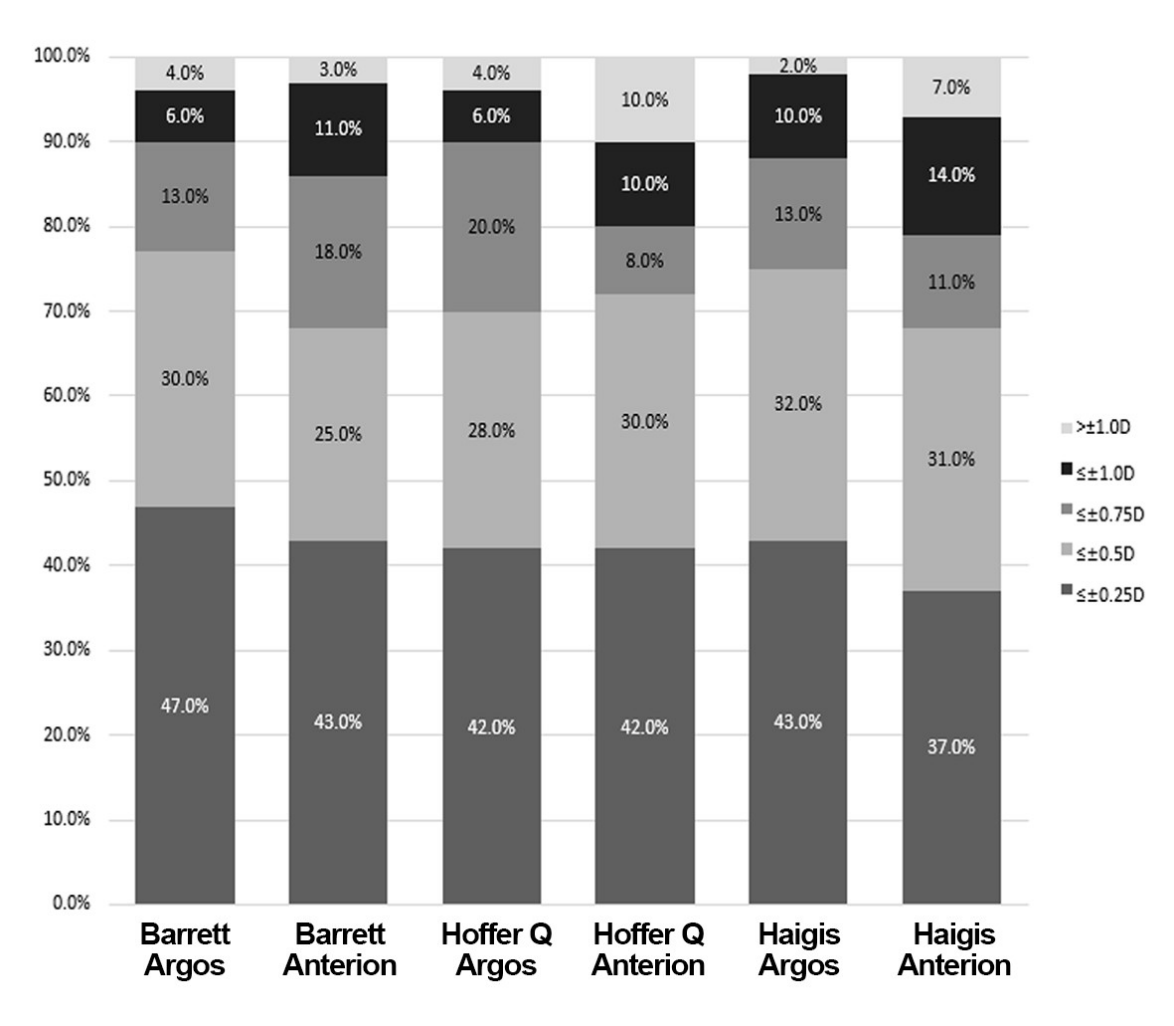
Stacked bar represents the proportion of eyes within a given diopter range of absolute prediction errors by the three formulas with Argos and Anterion.

In at least 70% of eyes evaluated using all formulas for Argos, the MAE did not exceed ± 0.50 D (Fig 2). The highest percentages were achieved with Barrett Universal II (77.0%), followed by Haigis (75.0%) and Hoffer Q (70.0%). Regarding the Anterion, the highest percentages were achieved with Hoffer Q (72.0%), followed by Barrett Universal II (68.0%) and Haigis (68.0%).

## Discussion

Various ocular measurement methods have been developed. While A-scan ultrasonography had long been the gold standard, it can underestimate AL by 0.1 to 0.3 mm as compared to immersion techniques due to its direct contact with the cornea [8,9]. Consequently, non-contact modalities, particularly PCI biometers, have become more prevalent than traditional ophthalmic echography [10]. However, the failure rate of PCI biometers was reported to be as high as 35.47% [11]. Failure has been reported to occur in instances of corneal opacity or severe posterior subcapsular cataracts attributable to compromised fixation or inhibited light transmission [12]. SS-OCT-based devices employ a light source with a longer wavelength than that those used for PCI, thereby enhancing cataract penetration and addressing these challenges [13]. In this study, we aimed to compare the biometric values and subsequent refractive outcomes of two SS-OCT-based biometers: Anterion and Argos.

Regarding the AL measurements, our findings presented results that are somewhat divergent from existing studies. Argos measured a shorter AL compared to Anterion (23.91 ± 1.63, Anterion vs. 23.89 ± 1.58 mm, Argos; p = 0.03); the mean difference was 0.02 mm. Considering that a 0.01-mm increase in AL reduces the IOL power by < 0.05 D, the discrepancy is of limited clinical insignificance [14,15]. In contrast, AL values measured by the Anterion were significantly shorter in studies by Schiano et al. as compared with the IOLMaster 500, and in those by Pfaeffli et al. as compared with the IOLMaster 700 [16,17]. The mean difference was 0.01 mm in both studies, which is clinically negligible in causing significant refractive error. According to Gjerdrum et al, there was no notable difference in AL measurements between Anterion and Argos (23.69 ± 0.96 vs. 23.69 ± 0.93 mm; p > 0.05). However, after categorizing patients into short (< 22.5 mm), normal (22.5–25.0 mm), and long (> 25.0 mm) eye groups, Anterion recorded statistically shorter AL in short eyes and longer AL in long eyes when compared to Argos [18]. Song et al. also showed no significant difference in the AL measurements between Anterion and IOLMaster 5.4 (23.78 ± 0.12 vs. 23.77 ± 0.12 mm, p > 0.05). Ultimately, while various studies have reported differences in AL measurements between the two devices, no clinically significant differences were found in terms of their impact on IOL calculation, which is consistent with our findings.

ACD serves as an indicator of effective lens positioning (ELP) following cataract surgery [19]. ELP can predict that anterior displacement of the IOL leads to myopia, whereas a posterior shift leads to hyperopic outcomes [20]. Thus, accurate ACD measurements are crucial for minimizing postoperative errors [21]. In our study, ACD demonstrated no significant differences between Anterion and Argos (3.14 ± 0.48, Anterion vs. 3.17 ± 0.45 mm, Argos; p = 0.11). Two prior studies observed a significant difference of 0.07 and 0.08 mm between Anterion and IOLMaster 700, respectively, but concluded that this variance was not clinically significant [17,22]. A shift of 0.07 mm in the ACD correlates to a refractive prediction deviation of < 0.08 D [23].

In this study, the Anterion showed significantly higher CCT and LT values than Argos. The mean differences were 4.09 um and 0.03 mm, respectively (p < 0.001 and p = 0.032), but these were not clinically significant. CCT has a minimal impact on traditional IOL calculation formulas. In terms of LT, Pfaeffli et al. reported comparable results with an additive offset of 0.07 mm. A previous analysis by Teshigawara et al. showed that such a discrepancy corresponds to a change in predictive refraction of < 0.2 D [17,23].

Additionally, we found statistically significant differences among the devices for CD, Kav, K1, and K2 (p < 0.001). Anterion reported shorter CD values and flatter keratometry readings as compared to Argos, with mean differences of -0.5 mm for CD, -0.23 D for Kav, -0.14 D for K1, and -0.33 D for K2. In relation to CD, this result was consistent with previous studies comparing the Anterion with the IOLMaster 700. The mean difference observed in our study was somewhat greater than that observed in other studies, but of little clinical relevance for IOL power calculation [17,23,24].

Regarding corneal curvature, Song et al. and Fisus et al. also demonstrated flatter values in the Anterion as compared to the IOLMaster 500 and 700, respectively [6,22]. In contrast, Pfaeffli et al. and Tana et al. found no significant differences between the Anterion and IOLMaster 700 [17,24]. In agreement with previous research, the disparities in measurement values between the two devices in our study can be attributed to the divergent methods of capturing the corneal curvature. Argos calculated the corneal curvature using OCT imaging at 2.2 mm and data derived from 16 projected light-emitting diode (LED) lights [25]. Conversely, Anterion employs 65-radial scans across a 3-mm zone for this purpose [6]. Given the aspheric nature of the cornea and its predominantly prolate shape, a larger measurement diameter is likely to yield a lower corneal power [16]. This methodological variance led the anterior to display a flatter corneal curvature, thus causing a hyperopic shift in target refraction and a myopic shift in ME.

In this study, the ME from the Anterion was more myopic than that from the Argos for all three IOL formulas (p < 0.05). This was consistent with the results of previous studies. According to Gjerdrum et al., the ME values were -0.14 ± 0.41 in the Anterion and 0.06 ± 0.41 in the Argos using the Barrett Universal II formula (p < 0.01) [16]. Song et al. demonstrated smaller ME values in the Anterion than in IOLMaster 5.4 using four different IOL formulas [6]. In the present study, the two devices showed good refractive predictability, with no statistically significant differences in MAE or percentage of eyes within +0.5 D of MAE, similar to the results of earlier studies [16,17].

The limitations of this study include its relatively small sample size and retrospective design. Considering that the Anterion utilizes a single refractive index, unlike Argos which uses segmental refractive indices, a subgroup analysis based on AL may have drawn noticeable results. The absence of a comparison with the widely used IOLMaster is another limitation. However, this shortfall might be mitigated by prior studies demonstrating strong inter-device repeatability between IOLMaster and Argos [26]. To the best of our knowledge, our study is the first to compare the Anterion and Argos, two SS-OCT devices, using various IOL formulas.

## Conclusion

In this study, comparison of the Anterion and Argos SS-OCT devices in IOL power calculations identified minor differences in measurement accuracy, with the Anterion showing a slight tendency towards more myopic refractive errors. These findings were consistent with those of previous studies showing reliable inter-device repeatability. This study provides novel insights by directly comparing SS-OCT devices using various IOL formulae.

## Supporting information

S1 Dataset (XLSX)
